# Supplementary material for: Modeling glioblastoma heterogeneity as a dynamic network of cell states
Source: Mol Syst Biol. 2021 Sep 16;17(9):e10105. doi: 10.15252/msb.202010105 (PMC8444284; doi:10.15252/msb.202010105)
Supplement: Supplementary file 6 — Source Data for Figure 5 [file MSB-17-e10105-s004.zip › Figure5A_sourcedata/GSEA_3017/nosigdb_stateE.GseaPreranked.1622795031093/LARSSON_STATE_4.html]

Details for gene set LARSSON\_STATE\_4[GSEA]

|  || Dataset | state63017 |
| Phenotype | NoPhenotypeAvailable |
| Upregulated in class | na\_neg |
| GeneSet | LARSSON\_STATE\_4 |
| Enrichment Score (ES) | -0.77259535 |
| Normalized Enrichment Score (NES) | -4.0754943 |
| Nominal p-value | 0.0 |
| FDR q-value | 0.0 |
| FWER p-Value | 0.0 |
Table: GSEA Results Summary

  

Fig 1: Enrichment plot: LARSSON\_STATE\_4      
 Profile of the Running ES Score & Positions of GeneSet Members on the Rank Ordered List

  

| PROBE | GENE SYMBOL | GENE\_TITLE | RANK IN GENE LIST | RANK METRIC SCORE | RUNNING ES | CORE ENRICHMENT || 1 | RAN |  |  | 298 | -0.257 | -0.3468 | No |
| 2 | MCM5 |  |  | 337 | -0.265 | -0.3879 | No |
| 3 | LSM4 |  |  | 406 | -0.284 | -0.4641 | No |
| 4 | SIVA1 |  |  | 412 | -0.286 | -0.4662 | No |
| 5 | HNRNPD |  |  | 456 | -0.301 | -0.5127 | No |
| 6 | MCM4 |  |  | 457 | -0.301 | -0.5087 | No |
| 7 | RFC2 |  |  | 498 | -0.313 | -0.5516 | No |
| 8 | LSM3 |  |  | 530 | -0.323 | -0.5837 | No |
| 9 | MCM3 |  |  | 560 | -0.340 | -0.6133 | No |
| 10 | LSM5 |  |  | 568 | -0.342 | -0.6170 | No |
| 11 | PHF19 |  |  | 648 | -0.391 | -0.7047 | No |
| 12 | GINS2 |  |  | 658 | -0.399 | -0.7100 | No |
| 13 | NUDT1 |  |  | 674 | -0.413 | -0.7222 | No |
| 14 | H2AFV |  |  | 684 | -0.423 | -0.7271 | No |
| 15 | RANBP1 |  |  | 697 | -0.427 | -0.7356 | No |
| 16 | ANP32B |  |  | 699 | -0.431 | -0.7311 | No |
| 17 | E2F1 |  |  | 708 | -0.435 | -0.7347 | No |
| 18 | ALYREF |  |  | 716 | -0.444 | -0.7371 | No |
| 19 | NUCKS1 |  |  | 744 | -0.471 | -0.7626 | No |
| 20 | PTMA |  |  | 745 | -0.475 | -0.7563 | No |
| 21 | TK1 |  |  | 747 | -0.480 | -0.7511 | No |
| 22 | GMNN |  |  | 766 | -0.510 | -0.7655 | No |
| 23 | CDC45 |  |  | 773 | -0.521 | -0.7657 | Yes |
| 24 | DTYMK |  |  | 774 | -0.522 | -0.7588 | Yes |
| 25 | STMN1 |  |  | 778 | -0.529 | -0.7553 | Yes |
| 26 | RPA3 |  |  | 788 | -0.550 | -0.7586 | Yes |
| 27 | HMGN2 |  |  | 795 | -0.564 | -0.7582 | Yes |
| 28 | HMGB1 |  |  | 797 | -0.567 | -0.7519 | Yes |
| 29 | ASF1B |  |  | 798 | -0.568 | -0.7444 | Yes |
| 30 | TUBB |  |  | 802 | -0.570 | -0.7404 | Yes |
| 31 | CENPN |  |  | 805 | -0.574 | -0.7352 | Yes |
| 32 | CHAF1A |  |  | 810 | -0.588 | -0.7321 | Yes |
| 33 | DEK |  |  | 812 | -0.591 | -0.7254 | Yes |
| 34 | MCM7 |  |  | 817 | -0.599 | -0.7222 | Yes |
| 35 | LMNB1 |  |  | 818 | -0.606 | -0.7142 | Yes |
| 36 | MND1 |  |  | 821 | -0.615 | -0.7084 | Yes |
| 37 | PCNA |  |  | 832 | -0.640 | -0.7117 | Yes |
| 38 | PIMREG |  |  | 833 | -0.641 | -0.7033 | Yes |
| 39 | CDKN3 |  |  | 836 | -0.653 | -0.6970 | Yes |
| 40 | BIRC5 |  |  | 843 | -0.679 | -0.6951 | Yes |
| 41 | CENPM |  |  | 844 | -0.682 | -0.6860 | Yes |
| 42 | BRCA1 |  |  | 846 | -0.691 | -0.6781 | Yes |
| 43 | ZWINT |  |  | 847 | -0.692 | -0.6689 | Yes |
| 44 | CENPH |  |  | 849 | -0.694 | -0.6609 | Yes |
| 45 | DHFR |  |  | 851 | -0.701 | -0.6528 | Yes |
| 46 | CENPK |  |  | 857 | -0.722 | -0.6492 | Yes |
| 47 | KIF4A |  |  | 861 | -0.736 | -0.6430 | Yes |
| 48 | FBXO5 |  |  | 864 | -0.742 | -0.6355 | Yes |
| 49 | RRM1 |  |  | 866 | -0.749 | -0.6268 | Yes |
| 50 | ORC6 |  |  | 870 | -0.766 | -0.6202 | Yes |
| 51 | TMPO |  |  | 875 | -0.778 | -0.6146 | Yes |
| 52 | PTTG1 |  |  | 880 | -0.791 | -0.6088 | Yes |
| 53 | MCM10 |  |  | 881 | -0.798 | -0.5983 | Yes |
| 54 | H2AFX |  |  | 882 | -0.799 | -0.5877 | Yes |
| 55 | EZH2 |  |  | 886 | -0.810 | -0.5805 | Yes |
| 56 | UBE2S |  |  | 887 | -0.815 | -0.5698 | Yes |
| 57 | SGO1 |  |  | 889 | -0.822 | -0.5601 | Yes |
| 58 | KIF15 |  |  | 890 | -0.834 | -0.5490 | Yes |
| 59 | H2AFZ |  |  | 893 | -0.849 | -0.5401 | Yes |
| 60 | RAD51AP1 |  |  | 894 | -0.853 | -0.5289 | Yes |
| 61 | KPNA2 |  |  | 895 | -0.858 | -0.5175 | Yes |
| 62 | DBF4 |  |  | 896 | -0.864 | -0.5061 | Yes |
| 63 | SMC2 |  |  | 897 | -0.867 | -0.4946 | Yes |
| 64 | SGO2 |  |  | 898 | -0.869 | -0.4831 | Yes |
| 65 | PBK |  |  | 900 | -0.880 | -0.4727 | Yes |
| 66 | FANCI |  |  | 901 | -0.882 | -0.4610 | Yes |
| 67 | SMC4 |  |  | 902 | -0.886 | -0.4493 | Yes |
| 68 | DUT |  |  | 903 | -0.887 | -0.4376 | Yes |
| 69 | CCNB1 |  |  | 906 | -0.895 | -0.4281 | Yes |
| 70 | CKS1B |  |  | 909 | -0.912 | -0.4184 | Yes |
| 71 | CKS2 |  |  | 911 | -0.922 | -0.4074 | Yes |
| 72 | GTSE1 |  |  | 912 | -0.941 | -0.3949 | Yes |
| 73 | MYBL2 |  |  | 914 | -0.959 | -0.3834 | Yes |
| 74 | CLSPN |  |  | 916 | -0.964 | -0.3719 | Yes |
| 75 | TYMS |  |  | 917 | -0.965 | -0.3591 | Yes |
| 76 | CENPU |  |  | 918 | -0.971 | -0.3463 | Yes |
| 77 | MAD2L1 |  |  | 921 | -0.990 | -0.3355 | Yes |
| 78 | UBE2T |  |  | 922 | -0.994 | -0.3224 | Yes |
| 79 | HIST1H1E |  |  | 925 | -1.049 | -0.3109 | Yes |
| 80 | ESCO2 |  |  | 926 | -1.067 | -0.2968 | Yes |
| 81 | UBE2C |  |  | 928 | -1.108 | -0.2833 | Yes |
| 82 | NUSAP1 |  |  | 929 | -1.112 | -0.2686 | Yes |
| 83 | TPX2 |  |  | 930 | -1.124 | -0.2537 | Yes |
| 84 | NCAPG |  |  | 931 | -1.142 | -0.2386 | Yes |
| 85 | ATAD2 |  |  | 933 | -1.162 | -0.2244 | Yes |
| 86 | CENPF |  |  | 935 | -1.200 | -0.2098 | Yes |
| 87 | HIST1H1C |  |  | 936 | -1.220 | -0.1936 | Yes |
| 88 | HMGB2 |  |  | 937 | -1.238 | -0.1773 | Yes |
| 89 | CDK1 |  |  | 938 | -1.248 | -0.1608 | Yes |
| 90 | RRM2 |  |  | 940 | -1.403 | -0.1434 | Yes |
| 91 | MKI67 |  |  | 941 | -1.407 | -0.1248 | Yes |
| 92 | TOP2A |  |  | 942 | -1.417 | -0.1060 | Yes |
| 93 | PCLAF |  |  | 943 | -1.554 | -0.0855 | Yes |
| 94 | HIST1H1D |  |  | 944 | -1.810 | -0.0616 | Yes |
| 95 | HIST1H1B |  |  | 945 | -1.903 | -0.0364 | Yes |
| 96 | HIST1H4C |  |  | 946 | -2.752 | -0.0000 | Yes |
Table: GSEA details [plain text format]

  

Fig 2: LARSSON\_STATE\_4: Random ES distribution      
 Gene set null distribution of ES for **LARSSON\_STATE\_4**
